# Increasing incidence and clinical burden of paediatric type 1 diabetes in Italy: insights from a large administrative database

**DOI:** 10.1007/s00592-026-02776-4

**Published:** 2026-08-21

**Authors:** Enza Mozzillo, Clara Trimarchi, Raffaella Buzzetti, Riccardo Candido, Valentino Cherubini, Basilio Pintaudi, Luca Degli Esposti, Melania Leogrande, Valentina Perrone, Chiara Capodaglio, Georgia Fousteri, Gian Paolo Fadini

**Affiliations:** 1 Department of Translational Medical Science, Paediatric Section, University Federico II, Napoli, Italy; 2 Sanofi, Milan, Italy; 3 Sapienza University of Rome, Rome, Italy; 4 Department of Medical Surgical and Health Sciences, University of Trieste, Trieste, Italy; 5 Department of Women’s and Children’s Health, Salesi Hospital, Ancona, Italy; 6 Niguarda Ca’ Granda Hospital, Milan, Italy; 7 CliCon S.r.l. Società Benefit, Bologna, Italy; 8 Department of Medicine, University of Padova, Padua, Italy

**Keywords:** Type 1 diabetes, Paediatric patients, Epidemiology, Autoimmune comorbidities, Healthcare resource utilisation, Administrative databases

## Abstract

**Background:**

Paediatric type 1 diabetes (T1D) is a chronic disease that represents a global public health concern. Italian data describing epidemiology, comorbidity burden, and healthcare resource utilisation (HCRU) of paediatric T1D remain fragmented, mostly deriving from regional registries or limited cohorts.

**Methods:**

A retrospective observational analysis was conducted using Italian administrative databases corresponding to ~ 12,000,000 individuals. Patients with T1D aged < 18 years were identified during 2010–2023 using diagnostic proxies (insulin prescriptions or diabetes-related hospitalisations). Epidemiological analyses were restricted to healthcare entities with continuous data availability during 2018–2022. Demographic and clinical characteristics were evaluated at baseline. Comorbidity onset, HCRU, and direct costs were assessed during follow-up.

**Results:**

A total of 3,220 paediatric patients with T1D were identified. Between 2018 and 2022, T1D incidence increased from 16.4 to 22.4 cases/100,000 individuals aged < 18 years (compound annual growth rate [CAGR] + 8.1%), while the prevalence reached 256 cases/100,000 in 2022 (CAGR + 10.0%). Most patients had minimal comorbidity at diagnosis. During follow-up, autoimmune conditions were the most common comorbidities, especially thyroid (8.9%) and coeliac disease (6.0%), often emerging within the first year post-diagnosis.

HCRU was the highest during the first year, with frequent diabetes-related hospitalisations and prescriptions of diabetes medications. Hospitalisation-related costs declined, whereas pharmaceutical costs increased over time.

**Conclusions:**

Paediatric T1D is an expanding chronic condition in Italy, characterised by early clustering of autoimmune comorbidities and substantial healthcare utilisation at diagnosis. These findings highlight the need for structured surveillance and coordinated care pathways to support long-term management and healthcare planning.

**Supplementary Information:**

The online version contains supplementary material available at https://doi.org/10.1007/s00592-026-02776-4.

## Introduction

Type 1 diabetes (T1D) is an autoimmune, progressive, and chronic disease that often develops in childhood and adolescence; it remains a major driver of long-term morbidity and healthcare resource utilisation (HCRU) [1–3]. Globally, T1D prevalence is increasing, with a substantial burden in younger populations [4]. The numbers of T1D are among the highest in several European countries, although data are not uniform. In Italy, available data are fragmented [5–9], with variable regional incidence ranging between 14 and 20 cases per 100,000 children and adolescents per year, and markedly higher rates in Sardinia [10].

The autoimmune nature of T1D is characterised by the presence of autoantibodies against pancreatic beta-cells: anti-glutamic acid decarboxylase autoantibodies (GADA), insulinoma-associated protein 2 autoantibodies (IA-2 A), insulin autoantibodies (IAA), zinc transporter 8 autoantibodies (ZnT8A), and islet cell cytoplasmic autoantibodies (ICA) [11–13].

Paediatric patients with T1D frequently present with, or later develop, additional autoimmune diseases, like thyroiditis and coeliac disease [14, 15]. Other comorbid conditions are also more common than in the general population [16–19].

Administrative databases represent a powerful tool to evaluate epidemiologic trends, quantify HCRU and related economic flows by integrating information on demographics, exemptions, prescriptions, hospitalisations, and outpatient services, thereby enabling the reconstruction of large-scale patterns of healthcare use [16, 17]. In Italy, detailed data describing the distribution and progression of healthcare costs among children with newly diagnosed T1D are limited [5, 18, 19].

The Administrative Italian Diabetes Analysis (AIDA) study was designed to provide updated real-world evidence and to support clinical and public health decision-making in the paediatric T1D population. The purpose of the analysis was to describe epidemiological trends, patient characteristics, comorbidities, HCRU, and related costs in children and adolescents with T1D in Italy.

## Methods

### Data source

A retrospective analysis was conducted using administrative records of geographically distributed Italian Local Health Units (LHUs), covering approximately 12.4 million individuals (about 20% of the Italian population. The analysis integrated several linked sources of administrative data, i.e. beneficiaries’ demographics, pharmaceutical, hospitalisation, outpatient specialist services (OSS), and exemption databases (Supplementary materials) [20] .

The dataset used consists of anonymised data. All the results of the analyses were produced and presented as aggregated summaries. Approval has been obtained from the ethics committees of the participating healthcare entities.

The dataset utilised by CliCon Società Benefit, an Italian research company specialising in real-world evidence, outcomes research, and health technology assessment using administrative and clinical healthcare databases. The data were anonymised at the source by the local health authorities that own them, through their own personnel or entities specifically appointed by the authorities themselves. Consequently, the data have already been processed by said local health authorities in the fulfilment of their institutional duties. Therefore, neither Article 110 of the Italian Privacy Code nor the provisions of the GDPR apply to the present analysis.

### Timeline

The study period spanned January 2009 to June 2024, with patient inclusion from January 2010 to June 2023. The index-date was the first evidence of T1D, corresponding to the earliest occurrence of diagnostic proxies. The characterisation period included all available data before the index-date, with at least 12 months of continuous data to ensure adequate baseline assessment. Follow-up extended from the index-date to the end of the observation window, with a minimum of 12 months. Only LHUs continuously present in the dataset between 2018 and 2022 were included for descriptive epidemiological analyses of T1D incidence and prevalence trends.

### Inclusion criteria

Patients aged < 18 years with T1D were identified through specific diagnostic proxies [9] during the inclusion period, namely: (i) at least one hospitalisation discharge diagnosis at primary or secondary level for T1D (ICD-9-CM: 250.x1; 250.x3); OR (ii) at least one prescription of insulins (ATC code: A10A).

### Exclusion criteria

Individuals with evidence suggestive of type 2 diabetes (T2D) were excluded, using prescriptions of glucose-lowering medications excluding insulin (ATC A10B) and/or hospitalisation with a primary or secondary diagnosis of T2D (ICD-9-CM 250.x0, 250.x2) as diagnosis proxies.

### Epidemiology data and trends

Incidence was defined as the annual number of newly identified T1D cases per 100,000 health-assisted individuals aged < 18 years. Prevalence was defined as the total number of pre-existing and new T1D cases per 100,000 health-assisted individuals aged < 18 years. Temporal trends were summarised using the compound annual growth rate (CAGR), calculated as (V_2022_/V_2018_)^1/4^ −1 × 100, where four represents the number of annual transitions between 2018 and 2022.

### Baseline characteristics

Age was reported in years and categorised into two groups (0–7 and 8–17 years); this cut-off was selected to distinguish younger children, whose diabetes management is predominantly caregiver-dependent and requires developmentally adapted approaches, from older children and adolescents [21]. The selected threshold is also supported by emerging evidence of age-related biological heterogeneity in T1D: disease onset in early childhood, particularly before approximately 7 years of age, is more frequently associated with T1D endotype 1 (T1DE1), which is characterised by distinct immunopathological features compared with T1D developing later in childhood or adolescence [22]. Sex distribution was described as the proportion of males. Baseline clinical status was evaluated using the Charlson Comorbidity Index (CCI), summarising overall comorbidity burden [23]. Although its performance has not been specifically assessed in paediatric T1D, the original CCI has been used in paediatric observational and claims-based studies [24–26]. In the present analysis, it was applied descriptively to provide a standardised summary of baseline comorbidity burden. Relevant conditions were recorded in the 12 months preceding the index-date, using medication records, hospitalisations, and exemption codes available in the administrative database; hence, patients who were untreated, not hospitalised, or without active exemptions for comorbidities were not captured.

The same criteria were used to detect comorbidities commonly associated with T1D, namely autoimmune thyroid disorders, coeliac disease, asthma, inflammatory bowel disease (IBD), rheumatic joint diseases, dermatological autoimmune disorders, and neurological conditions (i.e. epilepsy and cognitive impairment).

In the 12 months before index-date, drug prescription were summarised by ATC second-level classes, and hospitalisations by Major Diagnostic Categories (MDCs).

### Comorbidities during follow-up

The occurrence of concomitant conditions was monitored during follow-up, identifying incident cases from the index-date. Onset was classified as occurring in the first year, second year, or from the third year onwards. Time to onset was assessed using Kaplan–Meier cumulative incidence curves from the index-date to first occurrence, with censoring at the end of data availability.

### Antibody testing before T1D diagnosis

Islet autoantibody testing included assays for GADA, IA-2 A, IAA, ZnT8A, and ICA (procedure codes: 90.49.7, 90.50.5, 90.51.1), executed between 2018 and 2023. It should be noted that only tests recorded in the OSS database were captured, while tests performed during hospitalisations were untraceable. The presence of at least one test was assessed before the diagnosis of T1D. The timing of testing was evaluated by calculating the interval in months from the test date to the index-date. When multiple tests were present, the number of repetitions was quantified irrespective of antibody type.

### HCRU and direct costs

HCRU and cost analyses were conducted on patients with complete data for the corresponding period. Evaluations covered the first year of follow-up and annually up to five years thereafter. HCRU included medications, inpatient and day-hospital admissions, and outpatient laboratory tests, visits, and diagnostic procedures. Medical devices (i.e. glucose test strips, continuous glucose monitoring systems, insulin pumps, smart pens) were not captured because unavailable in the database. Mean annual costs per patient were calculated using NHS reimbursement tariffs. Values exceeding three standard deviations above the mean were treated as outliers and excluded, as they likely reflected a small number of patients with exceptionally high medical costs, potentially driven by severe acute complications, prolonged hospitalisations, or complex comorbid conditions, and could disproportionately influence mean cost estimates.

Diabetes-specific resources included antidiabetic medications, diabetes-related hospitalisations, and hospitalisations for hypoglycaemia.

### Statistical analysis

Descriptive statistics were conducted on continuous variables, given as mean with standard deviation (SD), and categorical variables reported as numbers and percentages.

According to Opinion 05/2014 on Anonymisation Techniques drafted by the European Commission Article 29 Working Party, analyses involving fewer than 3 patients were not reported, as they could potentially allow re-identification of single individuals. Therefore, results referring directly or indirectly to ≤ 3 patients were reported as NI (not issuable). All the analyses were performed using STATA SE version 17.0.

## Results

**Fig. 1:**
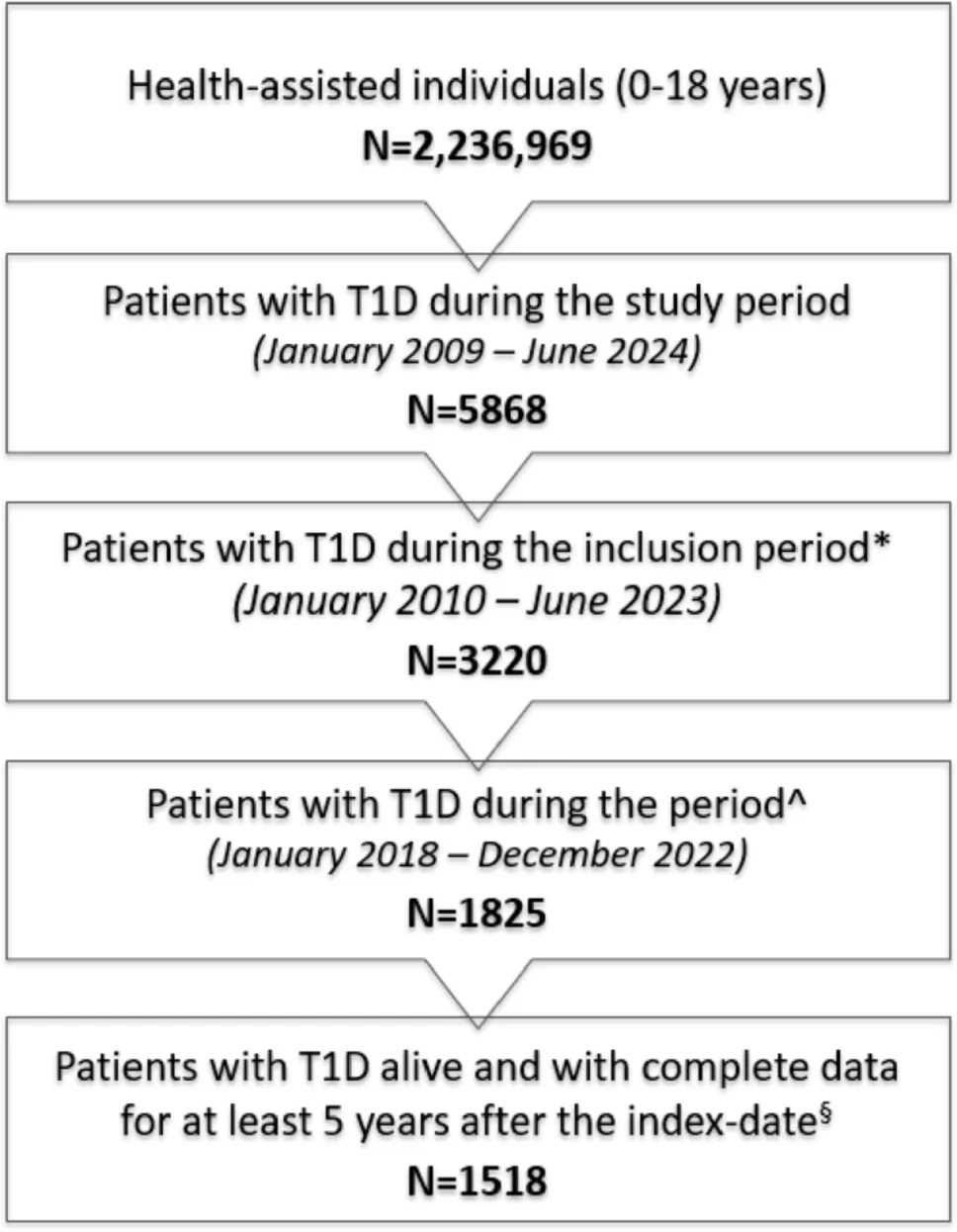
Flowchart of the selection of patients aged < 18 years included in the analysis. *Patients with 12 months of characterisation period and at least 12 months of data availability after index-date (the first evidence of T1D, corresponding to the earliest record of hospitalisation for T1D or first insulin prescription); ^Patients from LHUs with continuous data availability from 2018 to 2022 (included for the epidemiological analyses); ^§^Patients alive and with complete data for at least 5 years on whom analyses of HRCU and costs were performed. *T1D* type 1 diabetes

Among the 12.4 million health-assisted citizens, of whom 2,236,969 aged < 18 years, 3,220 paediatric patients were identified as having T1D based on diagnostic proxies and had at least 12 months of available data both before and after the first recorded evidence of T1D (i.e. the earliest occurrence of a hospitalisation with a T1D diagnosis, or a prescription for insulin). Of these, 1,825 children and adolescents from LHUs with continuous data availability for the period 2018–2022 were included in the analyses of epidemiological trends (Fig. 1).

### Epidemiology estimates

**Fig. 2:**
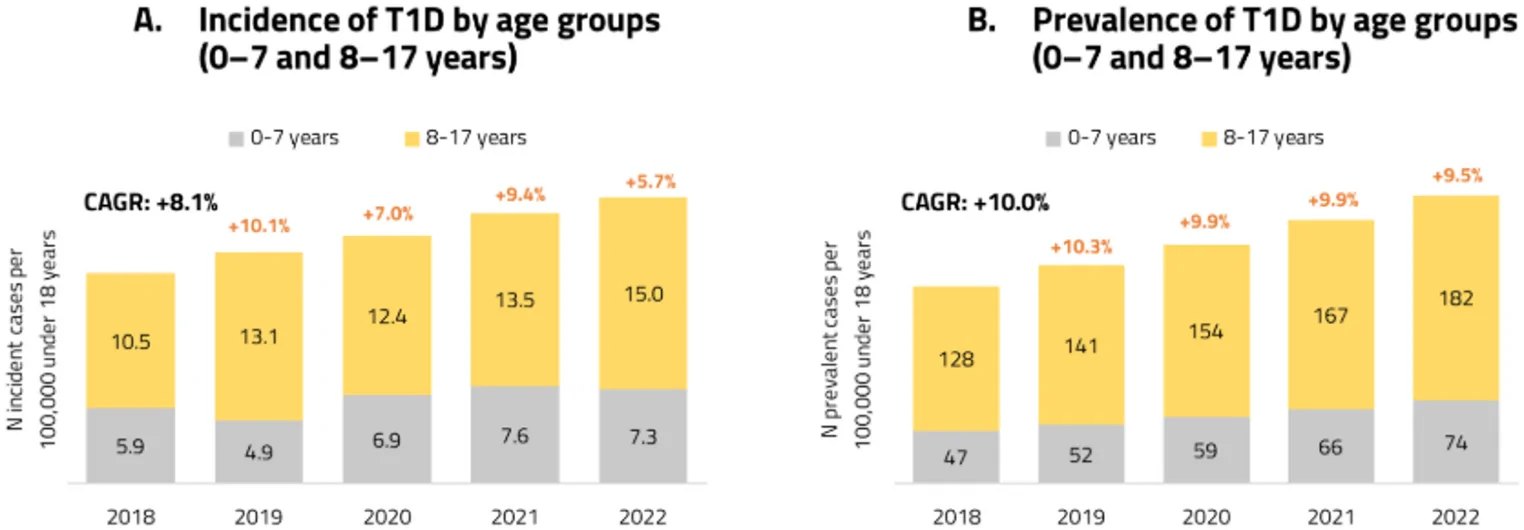
Epidemiology estimates of T1D (cases per 100,000 individuals < 18 years) over time (2018–2022): **A** incidence in patients stratified by age groups (0–7 and 8–17 years); **B** prevalence in patients stratified by age groups (0–7 and 8–17 years). *CAGR* compound annual growth rate, *T1D* type 1 diabetes

The annual incidence of T1D among individuals < 18 years rose across the period from 16.4 (2018) to 22.4 cases (2022) per 100,000 health-assisted (Fig. 2A). Detailed incidence rates with 95% confidence intervals are reported in Table S1 of the Supplementary materials. Among 1,825 new paediatric T1D cases identified, a CAGR of + 8.1% was observed. When clustered by age, incidence patterns were broadly consistent, with both age groups showing similar upward trends over time, although absolute rates remained slightly higher in the older patients).

Over the same period, the prevalence of T1D increased steadily, rising from 175 cases per 100,000 in 2018 to 193 in 2019, 213 in 2020, 233 in 2021, and reaching 256 cases per 100,000 in 2022 (Fig. 2B). Detailed prevalence rates with 95% confidence intervals are provided in Table S2 of the Supplementary materials. This corresponds to a CAGR of + 10.0% over the entire period. The upward trend was evident in both age groups, with prevalence rising among children aged 0–7 years and 8–17 years, although absolute rates were consistently higher in the older cohort due to the cumulative nature of disease burden.

### Demographic characteristics of children and adolescents with T1D

Among 1,825 patients with T1D aged < 18 years, the mean age at onset was 9.5 (4.4) years, specifically 4.2 years in children aged 0–7 years (*N* = 606), and 12.1 years in those aged 8–17 years (*N* = 1,209). Males accounted for 56% of the cohort, with similar proportions across age groups (data not shown).

### Clinical characteristics and comorbidities of children and adolescents with T1D

As Table 1 shows, in the overall population of 3,220 patients aged < 18 years with T1D (1,148 in the range 0–7 years and 2,072 in the range 8–17 years),: Comorbidity burden was generally low, with 81% of patients showing CCI = 0.

Thyroid disorders, coeliac disease, cognitive deficits, and epilepsy were the most frequently recorded conditions, though each affected fewer than 2% of patients at baseline.

Patterns of medication use reflected typical paediatric prescribing practices with antibiotics and respiratory medications being the most frequent drug classes, particularly among younger children (48.2% and 29.7%, respectively). Conditions related to the perinatal and neonatal period were more common in the younger subgroup, while distributions across other diagnostic categories were broadly similar.

**Table 1:** Main clinical characteristics of patients with T1D overall (< 18 years) and stratified by age (0–7 and 8–17 years)

	Patients with T1D, < 18 years (*N* = 3,220)	Patients with T1D, 0–7 years (*N* = 1,148)	Patients with T1D, 8–17 years (*N* = 2,072)
Baseline clinical characteristics			
CCI, mean (SD)	0.2 (0.4)	0.3 (0.5)	0.1 (0.4)
CCI = 0, N (%)	2,616 (81.2%)	813 (70.8%)	1,803 (87.0%)
CCI = 1, N (%)	583 (18.1%)	328 (28.6%)	255 (12.3%)
CCI ≥ 2, N (%)	21 (0.7%)	7 (0.6%)	14 (0.7%)
**Comorbidities during the characterisation period**,*** N (%)***			
Coeliac disease	61 (1.9%)	22 (1.9%)	39 (1.9%)
Thyroid disease	48 (1.5%)	7 (0.6%)	41 (2.0%)
Cognitive deficits	45 (1.4%)	15 (1.3%)	30 (1.4%)
Epilepsy	34 (1.1%)	NI	31 (1.5%)
Cystic fibrosis	14 (0.4%)	NI	13 (0.6%)
**Most frequent drugs in the 12-months before the index-date**,*** grouped by ATC***,*** N (%)***			
J01-Antibacterials for systemic use	1,204 (37.4%)	553 (48.2%)	651 (31.4%)
R03-Drugs for obstructive airway diseases	624 (19.4%)	341 (29.7%)	283 (13.7%)
H02-Corticosteroids for systemic use	262 (8.1%)	135 (11.8%)	127 (6.1%)
H04-Pancreatic hormones	214 (6.6%)	94 (8.2%)	120 (5.8%)
R06-Antihistamines for systemic use	170 (5.3%)	69 (6.0%)	101 (4.9%)
**Most frequent hospitalisations in the 12-months before the index-date**,*** grouped by DRG***,*** N (%)***			
Newborn and other neonates (perinatal period)	637 (19.8%)	422 (36.8%)	215 (10.4%)
Digestive system	179 (5.6%)	56 (4.9%)	123 (5.9%)
Ear, nose, mouth and throat	172 (5.3%)	51 (4.4%)	121 (5.8%)
Respiratory system	152 (4.7%)	67 (5.8%)	85 (4.1%)
Endocrine, nutritional and metabolic system	137 (4.3%)	41 (3.6%)	96 (4.6%)

*CCI* Charlson comorbidity index, *DRG* Diagnosis-Related Groups, *T1D* type 1 diabetes

### Occurrence of comorbidities during follow-up

After diagnosis, thyroid disease was the most frequent comorbidity, affecting 8.9% of overall paediatric patients, slightly more common in older children (9.5% vs. 7.8%). Coeliac disease was the second most common comorbidity (6.0% overall) and was more frequent in younger children (8.8% vs. 4.4%). Neurological conditions affected 2.6% of the patients. Asthma was identified in 2.2% of patients overall and was more common among younger vs. older patients (3.3% and 1.5%). Dermatological autoimmune diseases were rare.

Yearly analyses revealed that thyroid and coeliac disease occurred mostly in the first year of follow-up (58.7%). Conversely, neurological conditions were more evenly distributed over time, while asthma showed a later onset. Age-stratified analyses showed that first-year diagnoses of thyroid and coeliac disease were relatively more common in younger children. Neurological conditions occurred across follow-up in both groups, with earlier onset in younger and later onset in older children. Asthma showed a predominantly later onset. Kaplan–Meier cumulative incidence analyses indicated earlier onset of thyroid disease and coeliac disease after T1D diagnosis, whereas neurological conditions and asthma increased gradually over follow-up (Supplementary Figure S1).

### Autoantibody testing

#### Testing prescription and repetition

As reported in Table 2, before T1D diagnosis, 3.5% of patients had evidence of at least one autoantibody test prescription, with slightly higher testing rates observed among patients aged 8–17 years compared with those aged 0–7 years (4.1% vs. 2.4%). The interval between testing and diagnosis was short overall, with a median time of less than one month. Repeated testing before diagnosis was uncommon (0.3%). It should be underlined that the analysis of autoantibody testing was limited to assays recorded in the OSS database. Tests performed during hospitalisation could not be captured; therefore, the observed testing rates may underestimate the actual use of islet autoantibody testing in clinical practice. Available data suggest that testing occurred close to diagnosis, likely reflecting confirmatory use rather than early detection.

**Table 2:** Rates and timing of T1D-AAb testing before diagnosis of T1D in patients aged < 18 years, overall and stratified by age group (0–7 and 8–17 years). Testing was identified through OSS database

Before T1D diagnosis	Patients with T1D, < 18 years (*N* = 3,220)	Patients with T1D, 0–7 years (*N* = 1,148)	Patients with T1D, 8–17 years (*N* = 2,072)
Patients with at least one test, N (%)	112 (3.5%)	27 (2.4%)	85 (4.1%)
Time between test and diagnosis (months), mean (SD); median	3.7 (13.5); 0.2	2.4 (5.2); 0.3	4.1 (15.2); 0.2
Patients with more than one test (same or different), N (%)	10 (0.3%)	< 4	7 (0.3%)
Number of repetitions, mean (SD)	2.8 (0.9)	–	3.0 (1.0)

*T1D* type 1 diabetes

### HCRU and costs over five years of follow-up

Economic analyses were conducted on a subgroup of 1,518 patients with complete data for at least five years of follow-up (Table 3). The first year, including the index-date, showed the highest intensity of HCRU, as patients received an average of 5.2 diabetes-related drug prescriptions, with comparable values across age clusters. The use of non‑diabetes medications was lower, with a mean of 2.3 prescriptions per patient. As expected, hospitalisations related to diabetes were common during the first year, with an average of 1.13 admissions per patient, accounting for 60.3% of total hospitalisations. Among patients with at least one diabetes‑related hospitalisation, the average length of stay was 18.5 days. In the first year, among the 1,518 patients analysed, 451 (29.7%) experienced at least one hospitalisation for DKA. Among the 915 patients with at least one hospitalisation, this corresponded to 49.3% having at least one DKA-related admission. Other hospitalisations were rare, with a mean of 0.05 admissions per patient. OSS also contributed substantially, with an average of 3.4 OSS per patient.

**Table 3:** HCRU during the first year of follow-up (index-date included) in patients with T1D overall (< 18 years) and stratified by age (0–7 and 8–17 years) alive for at least five years

A. HCRU during the first year of follow-up (index-date included) Number per patient, mean (SD)	Patients with T1D, < 18 years (*N* = 1,518)	Patients with T1D, 0–7 years (*N* = 571)	Patients with T1D, 8–17 years (*N* = 947)
Number of dispensed diabetes-related drugs	5.2 (3.7)	5.0 (3.6)	5.4 (3.8)
Number of dispensed other drugs	2.3 (3.1)	2.8 (3.1)	2.0 (3.0)
Number of diabetes-related hospitalisations	1.13 (1.43)	1.24 (1.65)	1.06 (1.28)
Number of other hospitalisations	0.05 (0.29)	0.06 (0.29)	0.05 (0.29)
Number of OSS	3.4 (4.2)	2.8 (3.4)	3.7 (4.6)

*OSS* outpatient specialist services, *SD* standard deviation, *T1D* type 1 diabetes

The pattern of resource consumption was directly reflected in the observed cost profiles. The mean expenditure for drugs, hospitalisation, and OSS in the first year reached €1,905 per patient, with slightly higher costs in the 0–7 years age cluster (€1,938) compared with 8–17 years (€1,885).

**Fig. 3:**
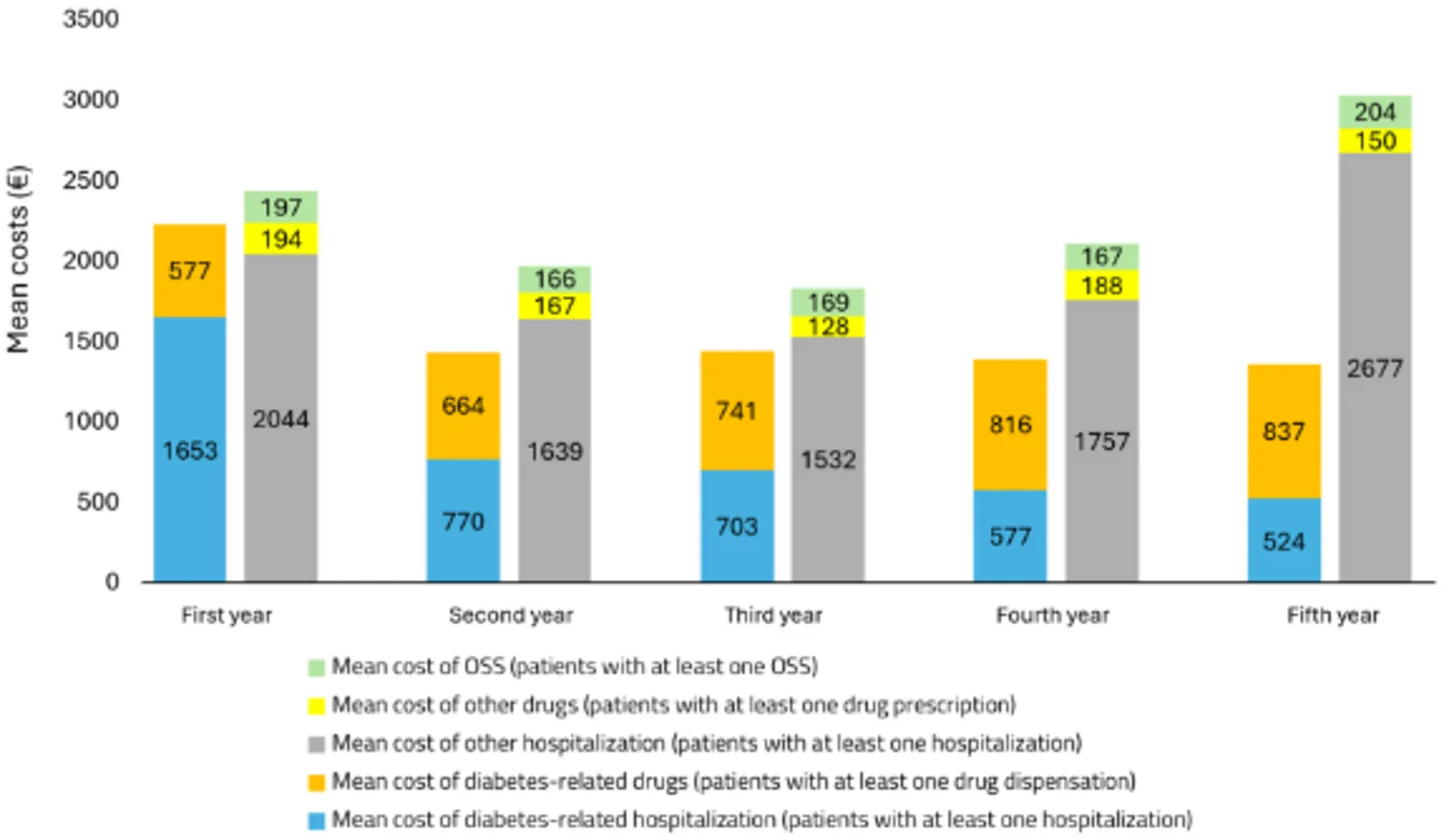
Mean annual NHS direct healthcare costs among patients with T1D with at least one claim during five years of follow-up, including diabetes-related hospitalisations, antidiabetic medications, other hospitalisations, other drugs, and outpatient specialist services (the costs for OSS did not include medical devices and diabetes technologies)

A longitudinal cost analysis was performed for each follow-up year among patients with at least one claim to quantify the economic impact on overall expenditures (Fig. 3). Mean costs of diabetes-related hospitalisations decreased steadily over time, from €1,652.73 in the first year to €523.75 in the fifth year. Conversely, costs for antidiabetic medications progressively increased from €576.93 in the first year to €836.82 by the fifth year. OSS expenditures remained relatively stable over time. Hence, among patients with at least one prescription for antidiabetic drugs, costs increased year over year through the fifth year (+ 15% in year 2, + 11.7% in year 3, + 10.1% in year 4, and + 2.6% in year 5). Conversely, among patients with at least one diabetes-related hospitalisation, costs declined over time (− 53.4% in year 2, − 8.7% in year 3, − 18% in year 4, and − 9.2% in year 5).

## Discussion

This analysis provides a real-world overview of paediatric T1D in Italy, integrating epidemiology, comorbidities, economic burden. Epidemiological estimates from 2018 to 2022 showed a consistent increase in paediatric T1D incidence, in line with data Italian National Institute of Health data [27] and regional cohorts, including Veneto [6], Calabria [28], Apulia [7], Piedmont and Marche [8]. Notably, population-based studies from Piedmont and Marche [8] and analyses from the Lombardy region [29] reported a marked increase during the COVID-19 period, following relatively stable trends in the previous years, consistent with patterns seen in Northern Europe [30]. The increase observed likely reflects both long-term trends and pandemic-related factors (e.g., delayed diagnosis, healthcare access changes, and possible SARS-CoV-2-related autoimmune mechanisms). Overall, these findings confirm a steady rise in incidence in Italy [9], consistent with global annual increase of 3–4% [2], and projections of growing T1D burden [4]. Although the reasons for the global increase are not fully understood, beyond genetic susceptibility, environmental and biological drivers, including viral triggers, metabolic factors, childhood overweight and obesity likely contribute, by promoting beta-cell stress and accelerate autoimmune destruction [31–35].

Thyroid and coeliac disease were the most frequently observed autoimmune comorbidities, with many new diagnoses within the first follow-up year, likely reflecting systematic screening and detection of previously subclinical disease [36–38]. Autoimmune thyroid disease is one of the most common comorbidities in childhood T1D and may arise at diagnosis or during follow-up [39–41] Coeliac disease is also frequent, with a prevalence of 6–16% depending on population and follow-up duration [37]. This is consistent with recent Italian population-based evidence showing a markedly increased prevalence and incidence of coeliac disease among children with T1D compared with the general paediatric population [42]. Age-related differences align with known autoimmune clustering patterns in T1D [40].

The analysis of autoantibody testing is limited to OSS-recorded assays [16]. Available data suggest testing occurred close to diagnosis, likely reflecting confirmatory use rather than early detection.

HCRU patterns revealed that the first year post-diagnosis was characterised by high hospitalisation rates and associated costs, driven by acute metabolic complications at onset, including diabetic ketoacidosis [18, 19], followed by a decline with clinical stabilisation. Concerning drug prescriptions, the higher use of antibiotics and respiratory medications may reflect both closer clinical monitoring and common paediatric prescribing patterns [43].

Insulin use showed a gradual and sustained increase over a five-year follow-up, consistent with the progressive decline of endogenous insulin secretion after diagnosis and with increasing insulin requirements during growth and puberty. Comparable trends have been observed in longitudinal clinical cohorts, which describe intensification of insulin therapy and increased use of basal–bolus regimens as children grow older [44]. This was paralleled by increasing pharmacological costs, consistent with treatment intensification, as recommended by Italian [45] and international standards of care [46].

A caveat of this cost analysis stems from the exclusion of medical devices, including glucose test strips, insulin pumps, and continuous glucose monitoring systems, has likely led to substantial underestimation of diabetes-related expenditures [17].

Reporting costs both for overall population and for patients with at least one claim represents an important methodological distinction, as analyses restricted to actual utilisers provide a more accurate per-user estimate [47]. The observed pattern—declining hospitalisation costs alongside stable or increasing pharmacological costs—mirrors findings from other paediatric diabetes studies and reflects a shift from acute, hospitalisation-driven expenditures to chronic therapy-related costs [19], consistent with the early “honeymoon phase” and subsequent treatment intensification [48].

Some limitations need to be acknowledged. Administrative databases are primarily designed for reimbursement rather than research, and lack detailed clinical information (e.g., BMI, HbA1c, DKA severity, or autoantibody levels). Moreover, T1D identification relied on administrative proxies (insulin prescriptions and/or hospitalisations), which may have introduced some degree of misclassification, potentially including other diabetes forms (e.g., MODY, insulin-treated T2D, or secondary diabetes). Although patients with T2D were excluded using A10B prescriptions and diagnostic codes, residual misclassification cannot be fully ruled out, as paediatric T2D may be insulin-treated or initially managed without non-insulin agents, and discordant coding may occur (e.g., T1D and T2D recorded in different hospitalisations). Moreover, the CCI was used as a standardised summary measure of baseline comorbidity burden. However, because the index was originally developed and validated in adult populations, it may not fully capture the age-specific comorbidity profile of children and adolescents with T1D. Although the conventional CCI has been applied in paediatric observational and claims-based studies [24–26], its validity and discriminatory performance have not been specifically established in paediatric T1D. Therefore, the CCI findings reported in the present study should be interpreted descriptively and with caution. Another point that deserves consideration is that glucose test strips, continuous glucose monitoring systems, insulin pumps, and smart pens were not captured in the database; their exclusion may therefore have led to an underestimation of the overall direct healthcare costs associated with paediatric T1D care. In addition, incidence estimates were based on the first recorded proxy and may reflect database capture rather than true clinical onset. Finally, although the dataset covers approximately 20% of the national population, it may not fully capture regional variation in disease burden. In particular, the results might suffer from the incomplete representation of all Italian regions, particularly the absence of Sardinia, which has one of the highest T1D incidence rates worldwide [10].

Despite these limitations, this analysis provides a comprehensive assessment of paediatric T1D epidemiology in Italy, integrating comorbidity and economic burden. The findings highlight the rising numbers of T1D, the clustering of autoimmune comorbidities, the high resource use at diagnosis, and the growing pharmacological needs with age among Italian children and adolescents.

In conclusion, paediatric T1D in Italy is an expanding condition characterised by rising incidence, early autoimmune comorbidities, and high initial healthcare utilisation, supporting the need for structured long-term surveillance and coordinated management.

## Supplementary Information

Below is the link to the electronic supplementary material.

Supplementary Material 1

## Data Availability

The data supporting the findings of this article are available at aggregated level from the authors upon reasonable request and with permission of the participating healthcare entities. Requests to access should be directed to corresponding author.
