# Supplementary material for: Increasing incidence and clinical burden of paediatric type 1 diabetes in Italy: insights from a large administrative database
Source: Acta Diabetol. 2026 Aug 21;63(11):1959–68. doi: 10.1007/s00592-026-02776-4 (PMC13690908; doi:10.1007/s00592-026-02776-4)
Supplement: Supplementary file 1 [file 592_2026_2776_MOESM1_ESM.docx]

**SUPPLEMENTARY MATERIALS**

**Detailed data source**

The following administrative databases were used for the analysis purposes:

- beneficiaries’ database, which included demographic information such as age, sex, and, when applicable, date of death;
- pharmaceutical database, which provided details on dispensed medications, including Anatomical Therapeutic Chemical (ATC) codes, prescription dates, and quantities supplied;
- hospitalisation database, which reported hospital admissions with corresponding dates, primary and secondary diagnoses coded according to the International Classification of Diseases, Ninth Revision, Clinical Modification (ICD-9-CM), as well as Diagnosis-Related Groups (DRGs);
- outpatient specialist services (OSS) database, which documented laboratory tests, diagnostic procedures, and specialist consultations, specifying the type and date of each service;
- exemption database which included payment-waiver codes that grant patients exemption from healthcare copayments in the presence of specific clinical conditions or diagnoses.
-

**Figure S1.** Schematic representation of the study timeline.

**Table S1.** Incidence rates with 95% confidence intervals (95% CI) of T1D per 100,000 health-assisted individuals aged <18 years, overall and stratified by age group (0–7 and 8–17 years).

| **Year** | **Incidence rate with 95% CI,**  **<18 years** | **Incidence rate with 95% CI,**  **0-7 years** | **Incidence rate with 95% CI,**  **8-17 years** |
| --- | --- | --- | --- |
| 2018 | 16.4 [14.6, 18.2] | 5.9 [4.8, 7.0] | 10.5 [9.1, 11.9] |
| 2019 | 18.1 [16.2, 19.9] | 4.9 [4.0, 5.9] | 13.1 [11.5, 14.7] |
| 2020 | 19.3 [17.4, 21.3] | 6.9 [5.7, 8.1] | 12.4 [10.9, 14.0] |
| 2021 | 21.1 [19.1, 23.2] | 7.6 [6.4, 8.8] | 13.5 [11.9, 15.2] |
| 2022 | 22.4 [20.3, 24.4] | 7.3 [6.2, 8.5] | 15.0 [13.3, 16.7] |

**Table S2.** Prevalence rates with 95% confidence intervals (95% CI) of T1D per 100,000 health-assisted individuals aged <18 years, overall and stratified by age group (0–7 and 8–17 years).

| **Year** | **Prevalence rate with 95% CI,**  **<18 years** | **Prevalence rate with 95% CI,**  **0-7 years** | **Prevalence rate with 95% CI,**  **8-17 years** |
| --- | --- | --- | --- |
| 2018 | 175 [170, 181] | 47 [44, 50] | 128 [123, 133] |
| 2019 | 193 [187, 200] | 52 [49, 55] | 141 [136, 147] |
| 2020 | 213 [206, 219] | 59 [56, 62] | 154 [148, 159] |
| 2021 | 234 [227, 240] | 66 [63, 70] | 167 [161, 173] |
| 2022 | 256 [249, 263] | 74 [70, 78] | 182 [176, 188] |

**Figure S2.** Kaplan–Meier survival analysis of time to onset of the most frequent concomitant conditions during follow-up among patients with T1D aged <18 years: (A) thyroid disease, (B) celiac disease, (C) cognitive deficits and (D) asthma.
